# From theory to autonomy: a topic modelling study of quantum finance through the lens of Datatopia and TOE

**DOI:** 10.3389/frai.2026.1761352

**Published:** 2026-03-11

**Authors:** Vasavi Durga Sesha Ratnam Desu, S. N. Vivek Raj

**Affiliations:** VIT Business School, Vellore Institute of Technology, Vellore, India

**Keywords:** AI in finance, Datatopia model, machine learning, natural language processing, quantum finance, structural topic modeling, text analytics, TOE model

## Abstract

Quantum finance is an emerging frontier that combines quantum theory with computational finance to deal with complex financial market dynamics. Despite of its rapid expansion, the field literature still remains fragmented, making it difficult to trace its intellectual development. So, Structural Topic Modeling, an AI-based unsupervised machine learning technique widely used in natural language processing, together with the Mann-Kendall trend test, to identify the latent thematic structure of the corpus and examine their temporal trends. Through STM, six topics are identified. The MK trend test is used for analyzing the temporal trends of articles collected based on PRISMA guidelines from both the databases, Scopus and Web of Science. The six identified topics are placed into the Datatopia quadrants, highlighting how the field spans from conceptually oriented “Aspiring Creativity” to the technologically autonomous, “Sorcerer’s Apprentice,” reflecting its expanding conceptual and technical foundations and the field’s growing alignment with AI-driven financial innovation. The TOE (Technology-Organization-Environment) framework further elucidates how technological advances, organisational adaptation and environmental factors shaped this transition toward AI-enabled financial innovation. The Mann–Kendall test showed positive thematic trends across AI-related topics, particularly “Quantum ML and Prediction,” while the final results also highlighted “algorithmic governance” as a new form of sustainability within AI-driven financial systems. Overall, this study provides a comprehensive view of the field’s development and demonstrates how combining topic modelling with conceptual frameworks can offer a systematic and scalable approach for analysing emerging interdisciplinary fields such as quantum finance.

## 1 Introduction

Financial markets have been expanding at a rapid rate in the last few decades and are increasingly dependent on advanced computational tools for risk management, derivative pricing and fraud detection. Traditional modeling approaches, however, have been challenged by the modern complex data environments. So, interdisciplinary approaches, particularly that integrate artificial intelligence, machine learning and quantum computational methods, are being used by researchers to overcome these challenges.

Because of this shift, quantum finance has emerged as a prominent frontier that combines quantum mechanics with computational finance. While class computers handle tasks step by step, quantum computers can utilise superposition and entanglement to process information in parallel. This makes them particularly effective for tasks like high-dimensional optimization and nonlinear modeling (Matwiejew et al., 2023; Pfeffer et al., 2022) supporting faster portfolio optimization, enhanced risk evaluation and innovative strategies to mitigate risk in dynamic markets.

As the field draws knowledge from many disciplines like quantum optimization, quantum machine learning, and quantum financial modeling, it results in fragmented literature. The fragmented literature makes it difficult to see the overall structure of the field and identify its emerging trends. In order to close this gap, the current work uses topic modeling to map changing research paths, examine publications in quantum finance, and give a more comprehensive view of the field’s evolution.

In order to study and comprehend the scholarly evolution of quantum finance, we have collected 81 documents from Web of Science and Scopus. There were no time constraints on the collection of the articles. To find the hidden themes and to examine how they evolved over time, Structural Topic Modeling, a topic modeling technique, was employed. This study is based on these research questions:

RQ1: What thematic patterns and recurring research topics emerge from the peer-reviewed literature on quantum finance?

RQ2: How do the prevalence and relative prominence of these research topics evolve over time within the quantum finance literature?

RQ3: What underexplored themes and conceptual tensions can be observed across the Datatopia quadrants, and how might these patterns be interpreted through the Technology–Organization–Environment (TOE) framework in quantum finance research?

The research questions are designed to guide a descriptive and exploratory analysis of the quantum finance literature rather than to enable statistical generalization.

The paper’s remaining structure is as follows: An overview of the current review articles is given in Section 2. The topic modeling approach employed in this work is described in Section 3, along with the model evaluation criteria. Section four includes the findings and analysis of topic modeling. Finally, Section 5 closes the work by summarizing the important contributions and offering directions for further research.

## 2 Related work

Over the last ten years, the growth has been particularly evident in the systematic reviews in the field of quantum finance. One systematic review has analyzed 94 publications that were published between 2001 and 2023 and categorized them according to research themes, data series, journals, and research techniques. The review paper identifies simulation, optimization, and machine learning are the key application domains in quantum finance (Liliana Bunescu, 2024). One more review study has analyzed all the studies that are published between 1952 and 2023 with an emphasis on blockchain applications, fraud detection, portfolio optimization, and Monte Carlo techniques for derivative pricing. It offered a two-fold examination of quantum algorithms and their uses in finance (Naik et al., 2025).

Narrative reviews, such as Dong et al. (2024), examine the development of the quantum finance field by highlighting its key theoretical frameworks, mathematical models, and algorithmic tools. Some of the reviews have focused on subfields like quantum machine learning in financial services by providing concise overviews of state-of-the-art algorithms and their potential in risk management, credit scoring, and fraud detection (Doosti et al., 2024).

Although these studies offered valuable insights, they have a narrow research focus and are lacking in a thorough and organized framework to examine the larger phenomena in the quantum finance literature. To identify latent themes and track research trends in the scientific literature of a field, the topic modeling method is employed for analyzing the large textual data (Gerlach et al., 2018).

STM is a computational text mining technique used to systematically review and analyze trends in quantum finance research. Unlike narrative reviews, which depend on subjective interpretation or bibliometric reviews that focus on publication and citation count, STM examines the entire textual corpus to identify latent research themes. Beyond text analysis, STM is distinguished from conventional topic modeling techniques such as LDA and NMF through the incorporation of document-level covariates such as publication year, author attributes and institutional affiliations into the model. These covariates help in enabling the analysis of the evolution of topics in different periods and contexts, thereby a deeper insight into quantum-enabled financial research is gained (Roberts et al., 2019).

The Datatopia framework has identified four alternative trajectories based on the dual dimensions of societal connectedness and the degree of technological control (Gartner, 2014). Aspiring Creativity is characterized by the environments where technological innovation is balanced by strong data governance and collaborative ecosystems. Society Inc. represents centralized, efficiency-driven systems with strong technological oversight and limited personal autonomy. Sorcerer Apprentice is represented by a rapid innovation environment that has surpassed regulatory and institutional frameworks, creating instability. Digital Wild West is represented by fragmented, low-trust environments in which technology operates without effective coordination or governance.

Collectively, these four scenarios describe how the information age could develop, from data utopian cooperative and privacy-respecting conditions to surveillance and chaos. Through this framework, the identified topics of the quantum finance field can be placed into the larger technological, organizational, and environmental (TOE) factors that influence the adoption of AI-enabled financial technologies. In this study, Datatopia is used as a conceptual and interpretive framework to contextualize STM-derived topics, rather than as a formal analytical or predictive model.

The TOE framework has been widely recognized as an effective model for analyzing technology adoption and diffusion. TOE has been applied in various research domains, including AI, blockchain, and enterprise applications, to assess how technological, organizational and environmental factors that have shaped the outcome of the adoption process (Badghish and Soomro, 2024; Ghaleb et al., 2021). While TOE explains the underlying operational and causal drivers of innovation diffusion, Datatopia contributes a structural and forward-looking perspective. When applied together, these frameworks connect meso-level adoption dynamics with macro-level future scenarios, thereby offering an integrated lens for studying AI-driven and data-intensive financial technologies.

## 3 Research methodology

### 3.1 Data collection

**Figure 1:**
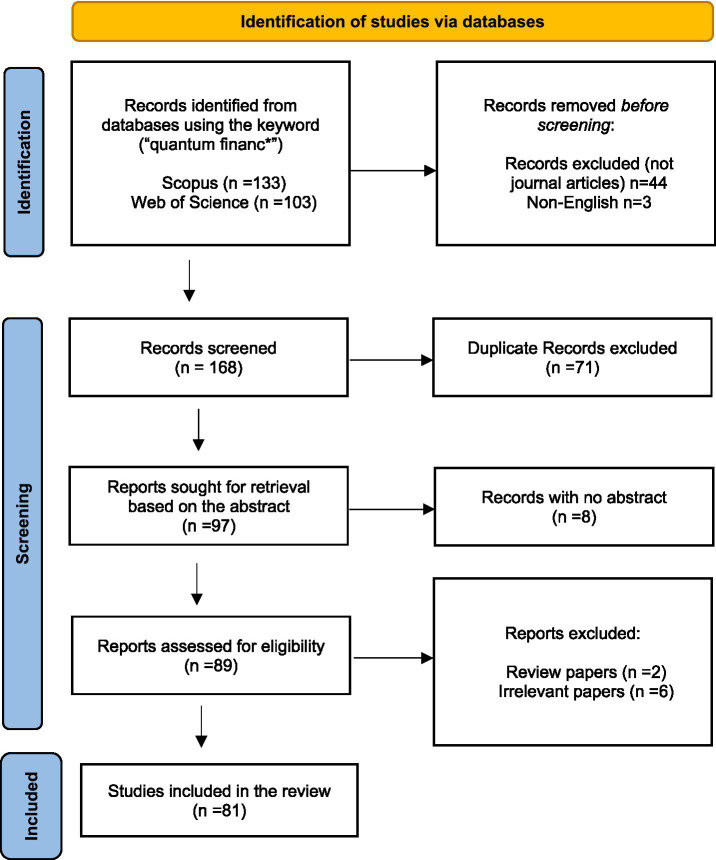
Prisma framework.

Scopus and Web of Science databases have been selected to identify and analyze academic articles on quantum finance due to their more comprehensive coverage, better data management, and advanced filtering options (Figure 1). A vast number of journals across all major scientific disciplines, including natural sciences, engineering, social sciences, and humanities has been indexed in both the databases. Scopus generally covers more journals, while Web of Science is more selective, often focusing on high-impact publications (Pranckutė, 2021; Singh et al., 2021). Together, these databases provide a balanced and reliable foundation for capturing established research patterns in the emerging field of quantum finance. The search string (“quantum financ*”) used for article retrieval is taken from an article (Liliana Bunescu, 2024).

We used the PRISMA framework (Page et al., 2021) to summarise the data collection process. The retrieved articles are checked for duplicates and screened for relevance. The systematic framework has helped us to select only relevant journal articles to be included in the dataset. As the present study is conceptual and methodological in nature, focusing on mapping research themes and trends rather than evaluating interventions or outcomes, the PICOS framework is not applicable and was therefore not employed.

### 3.2 Structural topic modeling

**Figure 2:**
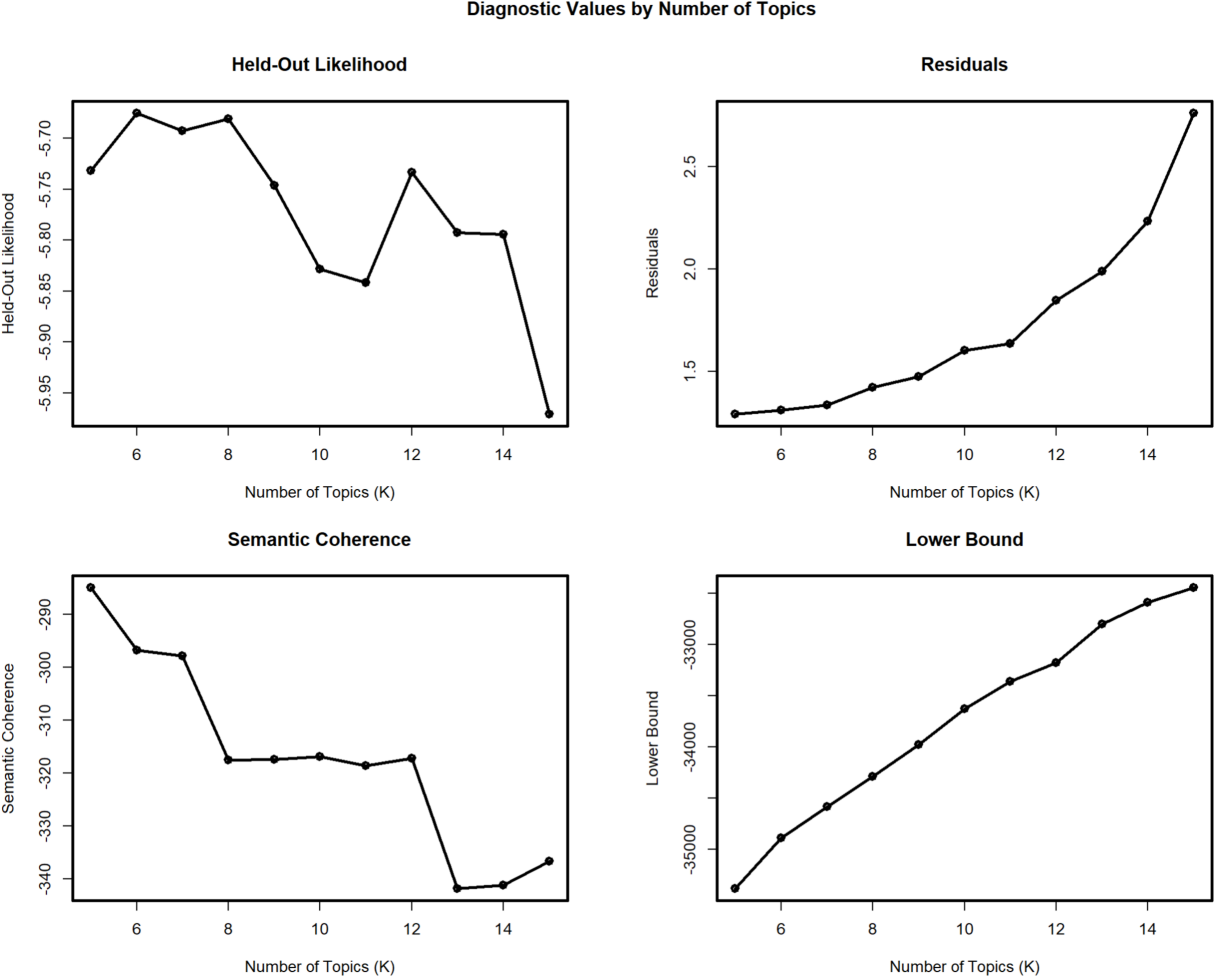
Identification of optimal number of topics (*k*).

STM was performed in RStudio. The preprocessing stage includes removing white spaces, lower case and custom stop words for data consistency. Then, the searchK function is used to identify the optimum number of topics. This function allows users to specify a range of topic numbers (k) and then evaluates each candidate model using multiple goodness-of-fit metrics (Weston et al., 2023). The held-out likelihood and semantic coherence are high at 6, thus providing that six is the optimum number of topics (Figure 2).

Semantic coherence and exclusivity are also measured in addition to model fit. These two metrics are widely used for evaluating the quality and interpretability of the extracted topics by determining whether topics are meaningful and are distinct from one another. Semantic coherence measures how frequently the top words in a topic co-occurred across documents, thereby reflecting the logical consistency and interpretability of a topic (O’Callaghan et al., 2015). In contrast, exclusivity is assessed by how unique the top words of a topic are when compared to those of other topics. A high exclusivity score was observed when a topic’s key terms are not shared with other topics, making each topic more distinct and interpretable within the model (Weston et al., 2023).

## 4 Results

### 4.1 Topic quality plot

**Figure 3:**
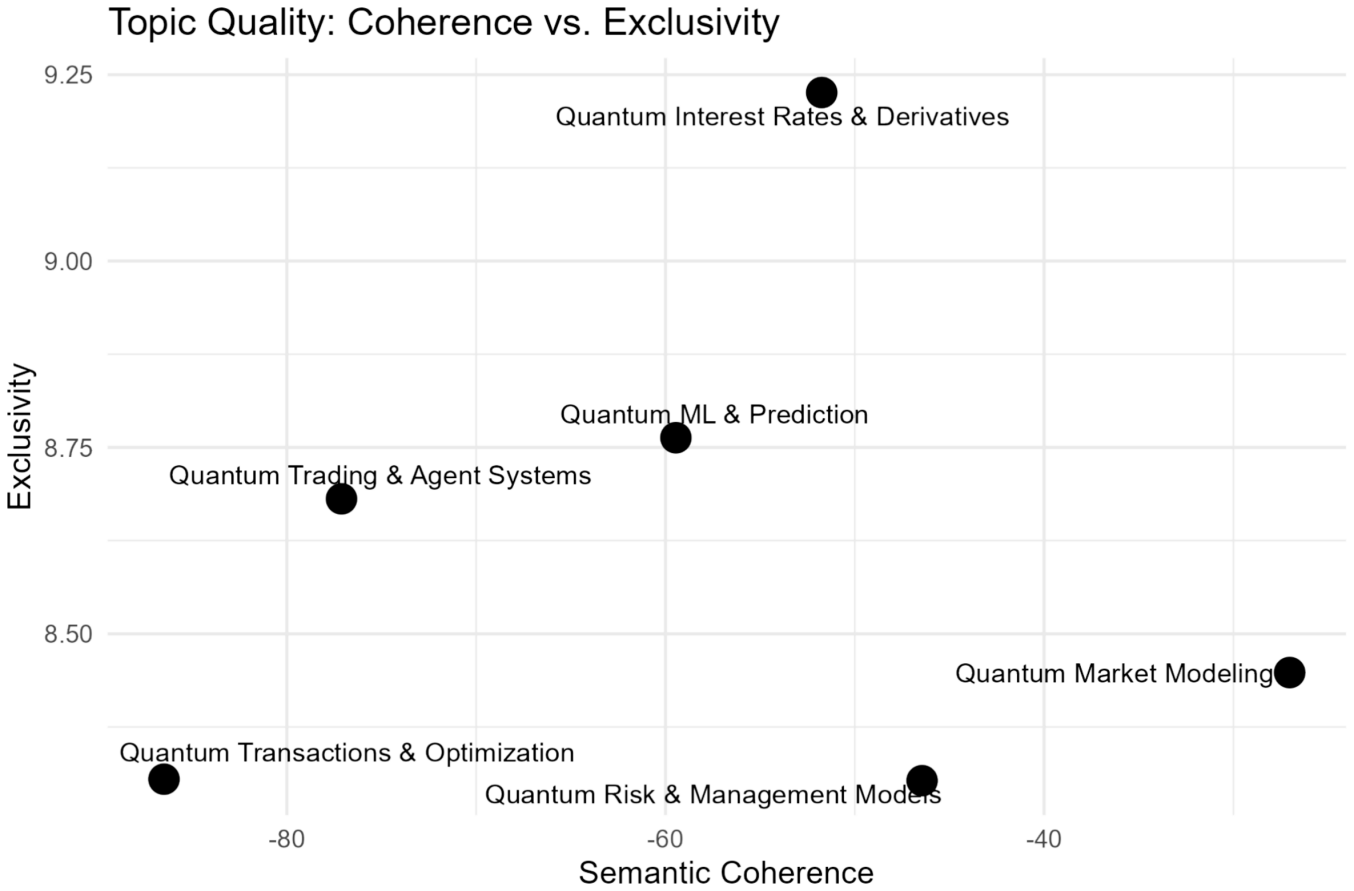
Topic quality plot.

The semantic coherence and exclusivity of the six topics were assessed in the topic quality plot (Figure 3). The distribution of topics across these dimensions has demonstrated that the selected model achieved a balanced representation of internally consistent yet thematically distinct topics. Topics with higher semantic coherence were associated with stronger term co-occurrence, whereas higher exclusivity values indicate clearer differentiation from other topics. Taken together, the plot suggests that the extracted topics are both interpretable and well defined.

The exclusivity values ranged from 8.26 to 9.21, with the topic “Quantum Interest Rates and Derivatives” identified as the most exclusive. The semantic coherence values varied between 88.09 to −26.06, and the topic “Quantum Market Modeling” exhibited the highest coherence score, indicating a strong cooccurrence pattern among its key terms. Negative coherence values are fairly common in topic modeling, meaning it is the degree of words appearing together in the documents (Airoldi and Bischof, 2016; Krasnashchok and Cherif, 2019). Overall, the plot helps us in achieving a balance between coherence and exclusivity.

We present the top 10 significant words for each topic based on FREX and High Prob in Table 1 for assigning the labels in a given document corpus. Together, these two metrics help in identifying the top words as FREX captures both frequency and exclusivity (Zhang et al., 2025; Weston et al., 2023). High Prob are very high-frequency terms that are commonly used to label the topics (Roberts et al., 2019).

**Table 1:** Identification of topic labels.

S_ No	Topic labels	Topic proportion	Highest probability words	FREX	Semantic coherence	Exclusivity	Top articles
1	Quantum Market Modeling	25.5	Financi, quantum, market, stock, price, model, reserv, right, elsevi, use, can, probabl	Stock, reserv, turbul, right, elsevi, evolut, probabl, oper, variabl, wave, financi, energi	−26.058	8.521	Cotfas (2013), Dima et al. (2015), Focardi et al. (2020), and Pedram (2012)
2	Quantum ML and Prediction	12.3	Quantum, model, use, classic, price, comput, data, predict, algorithm, state, market, learn	Train, load, advantag, classic, machin, predict, perform, state, qgan, trend, forecast, comput	−55.65	8.569	Alcazar et al. (2020, 2022), Manjunath et al. (2023), and Zoufal et al. (2019)
3	Quantum Risk and Management Models	14	Quantum, risk, financi, algorithm, comput, model, manag, studi, data, classic, propos, estim	Risk, manag, estim, analysi, assess, comput, enhanc, requir, explor, current, algorithm, limit	−49.105	8.285	Sivan and Priya (2025), Lin et al. (2024), Zafar (2025), and Zhou (2025)
4	Quantum Transactions and Optimization	13.7	Quantum, problem, paper, model, network, mechan, optim, use, payment, method, propos, financ	Problem, payment, transfer, fund, exchang, econom, network, effect, explain, consist, possibl, decis	−88.088	8.265	Meister and Price (2024), Orrell and Houshmand (2022), and Zheng et al. (2023)
5	Quantum Trading and Agent Systems	11.9	Trade, system, symmetri, quantum, learn, stock, agent, time, financ, indic, term, finance	Action, symmetri, agent, trade, fuzzi, invari, profit, deep, averag, reinforc, break, gradient	−80.33	8.842	Arraut et al. (2021) and Qiu et al. (2021, 2024)
6	Quantum Interest Rates and Derivatives	22.6	Rate, model, interest, price, option, quantum, libor, forward, bond, coupon, financ, market	Libor, bond, coupon, interest, swaption, rate, forward, lmm, formula, option, correl, propag	−51.748	9.214	Baaquie (2007), Baaquie and Liang (2007), and Kim et al. (2011)

### 4.2 Topic proportions

**Figure 4:**
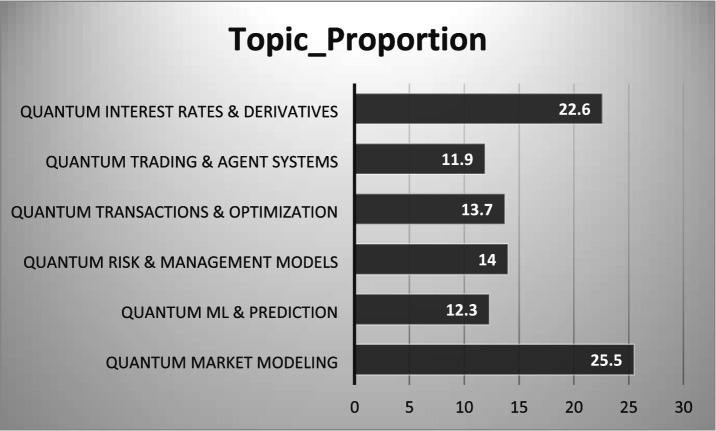
Topic proportions.

The relative importance of each topic is examined using topic proportion analysis, which represents the share of documents in the corpus associated with each latent theme (Figure 4). “Quantum Market Modeling” (25.5%) and “Quantum Interest Rates and Derivatives” at 22.6% have been identified as the most dominant topics in the corpus, together demonstrating the field’s strong base in theoretical and methodological orientation. The topics “Quantum Risk and Management Models” (14.0%) and “Quantum Transactions and Optimization” (13.7%) are in the middle range, suggesting an increasing interest in quantum-enabled optimization and risk analysis. The remaining topics, “Quantum Machine Learning and Prediction” (12.3%) and “Quantum Trading and Agent Systems” (11.9%), are appearing as emerging fields, where quantum computing is being combined with AI-enabled financial applications.

### 4.3 Mann Kendall trend

The Mann–Kendall test is recognised as a robust, non-parametric test for identifying monotonic trends in time series data. The test evaluates whether a topic’s prominence shows a consistent increasing or decreasing pattern over time without requiring assumptions about data normality. The test uses Kendall’s Tau (*τ*), a correlation coefficient to capture both the strength and direction of the trend. The values of τ range from −1 (strong decreasing trend) to +1 (strong increasing trend), and values near 0 indicate no clear, consistent trend (Brossart et al., 2018; Faquseh and Grossi, 2024; Takin et al., 2024). The *p*-value associated with *τ* helps in determining the statistical significance, and trends are usually considered significant when *p* < 0.05 (Chen et al., 2022; Faquseh and Grossi, 2024).

To verify the independence assumption underlying the Mann–Kendall test, we examined first-order (lag-1) autocorrelation in yearly topic prevalence series. Lag-1 autocorrelation coefficients were small and statistically insignificant for all topics, indicating the absence of meaningful serial dependence as reported in Table 2.

**Table 2:** Mann–Kendall trends and Lag-1 ACF values of the identified topics.

Topic	Kendall τ	*p*-value	ACF (Lag 1)	Autocorrelated
Quantum Market Modeling	−0.216	0.208	−0.047	False
Quantum ML and Prediction	0.462	0.006	0.188	False
Quantum Risk and Management Models	0.696	0.000	0.034	False
Quantum Transactions and Optimization	0.415	0.014	0.124	False
Quantum Trading and Agent Systems	0.462	0.006	0.344	False
Quantum Interest Rates and Derivatives	−0.602	0.000	0.237	False

The four topics “Quantum ML and Prediction,” “Quantum Risk and Management Models,” “Quantum Transactions and Optimization,” and “Quantum Trading and Agent Systems” have positive *τ* values, indicating clear, significant upward trends (Table 2). This indicates growing research interest in data-driven, computational, and autonomous systems in quantum finance. The two topics “Quantum Market Modeling” and “Quantum Interest Rates and Derivatives” have negative τ values. “Quantum Market Modeling” has a stable or no significant trend, and “Quantum Interest Rates and Derivatives, shows a significant downward trend, reflecting a significant decrease in attention. This shows that border theoretical modeling remained stable due to its wider applicability, but specialised domain, quantum interest rates and derivative modeling is losing momentum because of the limited practical advantage after industry reforms.

### 4.4 Clustering

**Figure 5:**
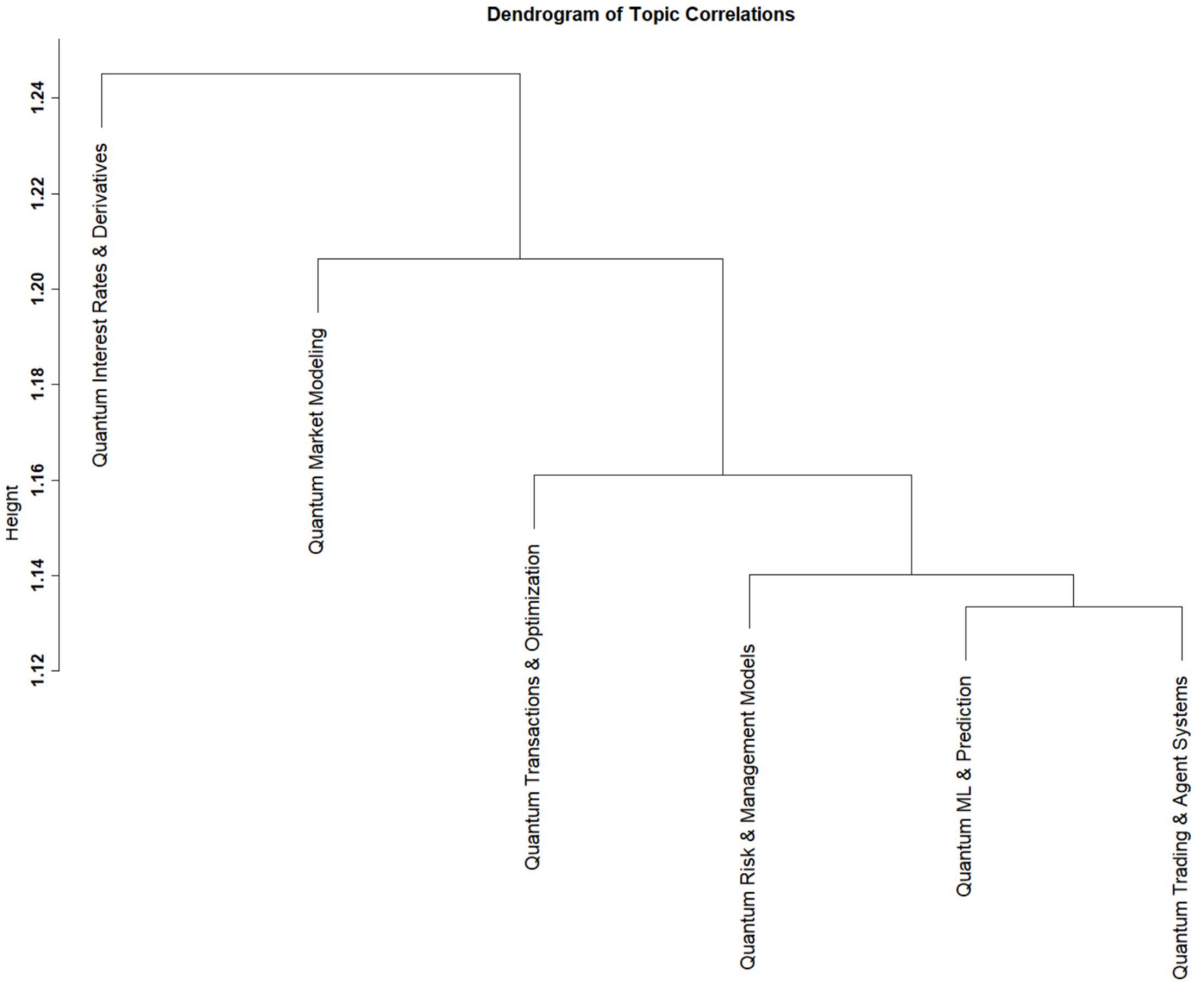
Clustering of the topics.

The dendrogram (Figure 5) reveals four distinct topic clusters based on the correlation structure among STM topics (Jeyasree et al., 2022; Zhao et al., 2014). The first cluster has a single topic, “Quantum Interest Rates and Derivatives.” The second cluster includes “Quantum Market Modeling.” The third cluster has two topics, which are “Quantum Transactions and Optimization” and “Quantum Risk and Management Models,” a combination of risk management, portfolio design, and decision-making. Lastly, the topics in the fourth cluster are “Quantum ML and Prediction” and “Quantum Trading and Agent Systems,” the two topics that combine quantum techniques with AI. The four clusters were further mapped to the four quadrants of the Datatopia model to determine future directions in quantum research based othe Datatopia-TOE framework.

### 4.5 Datatopia model with TOE framework

#### 4.5.1 Quadrant 1: Society, Inc.—“whatever”

In this quadrant, “Quantum Modeling of Interest Rates and Derivatives” is emphasized, covering LIBOR and EURIBOR models (Baaquie and Yang, 2009) to bond and option pricing approaches (Baaquie and Liang, 2007). It represents a mature, application-oriented stage within the quantum finance research domain. Work in this area is simulated by real market data and industry standards. The pricing and hedging strategies are formulated in the regulated financial environments.

The environmental dimension of the TOE framework plays a major role in this quadrant. Regulatory requirements and validation protocols of institutions play a major role. In the Datatopia model, the topic matches with the “Society, Inc.” quadrant because here, technological innovation is controlled in a competitive marketplace and “conflict” arises from the tension between adherence to standards and innovation. The quantum models are operated under institutional oversight and strict compliance requirements.

#### 4.5.2 Quadrant 2: Datatopia “aspiring creativity”

The foundational topic “Quantum Market Modeling” falls within this quadrant. This quadrant is a core of quantum finance research technology. In this quadrant, research studies are largely motivated by the use of quantum mechanics in the financial modeling. Such studies include more advanced conceptual models, like Schrodinger-based price dynamics (Dima et al., 2015), quantum probability option pricing (Focardi et al., 2020), and finite-Hilbert-space models (Cotfas, 2013). It is the quadrant which is driven by innovation arising out of inquiry and not out of external factors such as market or policy demands. Here, the institutions are not promoters of innovation but facilitators. Aspiring Creativity is another related yet controlled space in which the interdisciplinary cooperation orients the quantum technologies to design innovations. In this regard, technology promotes creative theorization in line with new computational abilities in the actual world. In this quadrant, the dominant TOE driver is technology, as innovation in research is led by theoretical advances rather than organizational or environmental factors. The topic “Quantum Market Modeling” fits in this quadrant is because this topic is exploratory, innovation-driven, and its progression is led by technological innovation and inquiry rather than organisational or environmental pressures.

#### 4.5.3 Quadrant 3: Digital wild west “state of the nature”

The “Quantum Transactions & Optimization” and “Quantum Risk and Management” topics are in this quadrant. This quadrant represents a transitional and experimental space in quantum finance. Research in this quadrant combines payment network modeling, transaction settlement (Zheng et al., 2023), and game-based optimization (Meister and Price, 2024) under emerging quantum computational methods. The TOE dominant driver in this quadrant is technology. The research in this area is primarily focused on developing and testing new algorithms in a fragmented, low-control environment with limited standardization, which is in contrast to institutionally regulated financial modeling. Within the Datatopia framework, this pattern was aligned with the Digital Wild West quadrant, which is characterized as a conflicted and creative environment. Here, the “conflicted” nature has come from the fragmented nature of technology marked by many overlapping experiments. Such fragmentation has created a productive environment, allowing innovation to progress rapidly with minimal regulatory control. These topics are positioned here because their advancement is driven by technological innovation in unstructured environments that have consistently outpaced institutional adaptation or establish shared frameworks.

#### 4.5.4 Quadrant 4: Sorcerer’s apprentice “ruled by machines”

In our analysis, the emerging topics “Quantum ML & Prediction” and “Quantum Trading and Agent Systems” are placed in this quadrant. The Sorcerer’s Apprentice category was characterized by the rapid emergence of quantum-AI integration in financial modeling, where autonomous algorithms operate within highly interconnected digital environments. Research within this area has been represented by the quantum finance-reinforcement learning hybrids (QF-FRL, QFPIS), deep neural trading agents (QF-TraderNet) (Qiu et al., 2021, 2024), and hybrid quantum-classical frameworks (QGAN, QCBM, QNN) (Alcazar et al., 2020; Zoufal et al., 2019). These models together demonstrated that quantum systems are capable of self-correction, adaptive decision making, and perform unsupervised optimization, indicating a shift toward algorithmic autonomy. Technology has been identified as the dominant TOE element, with innovation acting as the main driver. The Sorcerer’s Apprentice quadrant is described as a connected yet chaotic environment where quantum systems engage with each other in an interconnected realm, while “amok” indicates that they have developed beyond the realm of human oversight. These topics are positioned in this quadrant because their progression is relied on autonomous AI-driven quantum systems whose capabilities have developed faster within highly connected environments, surpassing any human control (Table 3).

**Table 3:** Datatopia model with the topics (Gartner, 2014; Shukla et al., 2024).

		Society/World	
		Conflicted (In the conflicted world, technologies are often used to block other technologies. Goals are conflicting.)	Connected (In the connected world, all kinds of technologies interact. Goals are aligned.)
Technology	Control (In the controlled world, we know what we want technology to do and make it so)	Quadrant 1: Society, Inc. Whatever Topics: “Quantum Interest Rates and Derivatives”	Quadrant 2: Datatopia Aspiring Creativity Topics: “Quantum Risk Modeling”
	Amok (When technology runs amok, society does nothing but respond)	Quadrant 3: Digital Wild West State of the Nature Topics: “Quantum Transactions and Optimization” “Quantum Risk Management and Models”	Quadrant 4: Sorcerer’s Apprentice Ruled by Machines Topics: “Quantum Trading and Agent Systems” “Quantum ML and Prediction”

## 5 Discussion

To answer RQ1, STM was applied to identify six dominant topics in the quantum finance literature. The topics are “Quantum ML and Prediction,” “Quantum Risk and Management Models,” “Quantum Transactions and Optimization,” “Quantum Trading and Agent Systems,” “Quantum Market Modeling” and “Quantum Interest Rates and Derivatives.” “Quantum Market Modeling” (25.5%) and “Quantum Interest Rates and Derivatives” (22.6%) account for the largest share of publications, signifying their prominence in the quantum finance literature.

To answer RQ2, we conducted an MK trend test to study the temporal trends of these topics. The results show a significant increasing trend in topics “Quantum ML and Prediction,” “Quantum Risk and Management Models,” “Quantum Transactions and Optimization,” Quantum Trading and Agent Systems,” indicating a rising scholarly interest in application-oriented and computationally feasible research streams. In contrast, “Quantum Market Modeling” exhibits no statistically significant trend and “Quantum Interest Rates and Derivatives” exhibits a statistically significant declining trend, suggesting theoretical maturity. The decline is attributed to the hardware limitations and computational constraints, like the inability to process negative eigenvalues and the qubit capacity required for the complex derivative pricing (Chakrabarti et al., 2021; Naik et al., 2025). In addition to this, the field is also shifting to feasible applications such as optimization and quantum AI, which are more achievable in the near future (Liliana Bunescu, 2024).

Overall, the transition in trends indicate that the field is shifting from abstract quantum models to a computationally autonomous phase, characterized by machine learning and quantum algorithms for forecasting, risk analysis and trade optimization.

In response to RQ3, we used the Datatopia–TOE mapping to reveal the future trajectories of the six STM topics. This future-oriented approach helps us in identifying the emerging gaps and outlining the future directions for each Datatopia quadrant:

### 5.1 Quadrant 1: Control-conflicted

In the “Society, Inc.” quadrant, quantum finance research operates primarily on interest rate and derivative modeling. This quadrant is a mature, compliance-driven environment, dominated by environmental and regulatory forces. However, strong regulatory constraints tend to limit innovation, as researchers often remain within strict compliance boundaries (Fu et al., 2022; Jiang et al., 2022). As a result, many quantum studies rely on extensions of classical pricing logic models, rather than experimenting with new quantum approaches that are adaptive to new markets and policy regimes (Gómez et al., 2022). Moreover, few studies have considered issues of transparency, credibility and accountability in the application of quantum systems to finance (Liliana Bunescu, 2024). Addressing these issues will help us build long-term sustainability in the field.

Based on Datatopia, future research is expected to balance between compliance and innovation by developing quantum derivative models that are both regulatorily robust and technologically adaptive. In particular, studies could explore DeFi-based quantum systems and add ethical auditing frameworks to improve trust and accountability (Mökander and Floridi, 2021). From a TOE perspective, progress in technological capabilities through better quantum-based pricing models, better organisational practices in framing clear ethical and audit processes and a supportive regulatory environment can help in transitioning this quadrant from a regulation-bound space to a more innovation-friendly ecosystem.

### 5.2 Quadrant 2: Control-connected

In the “Aspiring Creativity” quadrant, research is primarily shaped by technological innovation originating from quantum foundations and mathematical modeling, leading to the formulation of conceptually strong theories. Most of the research work is confined within the academic boundaries, resulting in many theoretical models. Consequently, there is a clear application gap, because only limited models are tested with real market data (Coyle et al., 2021; Liliana Bunescu, 2024) and there is also an integration gap between abstract modeling and practical financial uses, such as portfolio management or trading strategies.

The quadrant is characterized as controlled, as research progresses in a guided and methodological way, and is also connected due to the interdisciplinary collaboration across physics, mathematics and finance working toward a common objective.

Based on Datatopia, future research should focus on linking theoretical precision with market validation, developing quantum–classical testing frameworks, and building interdisciplinary teams that can shift abstract models to practical market intelligence to advance this stream (France and Ghose, 2019). From a TOE perspective, advances in technology (hybrid testing pipelines), a supportive ecosystem for experimentation and organisations enabling interdisciplinary research teams can collectively facilitate the theory-driven research to more applied, data-driven innovation.

### 5.3 Quadrant 3: Conflicted + amok

In the “Digital Wild West” quadrant, quantum finance research focuses on quantum transactions, payment networks, and optimization algorithms, with innovation growing faster than institutional readiness to respond. The research in this quadrant is highly experimental and rapidly evolving driven primarily by technological enthusiasm and is hampered by organizational fragmentation in the field.

As a result, a standardization gap is created, as there are no widely accepted performance metrics for comparing quantum optimization methods in financial applications, hindering the replication of results (Lubinski et al., 2024). A related governance gap is also evident, since there are no clear rules for governing quantum-based financial applications, leading to systemic risks. The absence of safeguards for fairness and risk control is further amplified by the limited infrastructure and, shortage of professionals with quantum expertise also led to an organizational readiness gap (Kaur and Venegas-Gomez, 2022).

Based on Datatopia, future research should aim to bring common performance standards, develop regulatory and governance models for quantum algorithmic trading (Lee and Schu, 2022), and also assess institutional readiness through empirical and longitudinal studies. To transition a fragmented experimentation into a scalable, trustworthy, and sustainable framework, a more coherent and accountable ecosystem has to be built for quantum financial innovation. From a TOE perspective, technological progress like better benchmarking tools and more secure quantum pipelines, improving organisational capacity, and environmental guardrails that can clarify responsibilities are essential for transforming this quadrant from a fragmented experimentation-driven space to a more coordinated and trusted innovation-ready ecosystem.

### 5.4 Quadrant 4: Connected + amok

In the “Sorcerer’s Apprentice” quadrant, quantum finance research converges with artificial intelligence to create autonomous trading and decision systems that operate within dense, high-speed hybrid networks to enhance trading performance and portfolio optimization.

Interpreting and monitoring the algorithmic activities in quantum-enhanced trading markets is increasingly challenging due to the growing self-learning capability of quantum AI systems (El Hajj and Hammoud, 2023). At the same time, fairness, accountability, and the development of regulatory frameworks for regulating the quantum financial markets are not receiving enough attention, resulting in a growing ethical and institutional gap.

Based on the Datatopia framework, future research should develop explainable quantum–AI systems and transparent quantum AI model architectures that maintain human-in-the-loop supervision (Retzlaff et al., 2024). In parallel, scholars should also explore ethical governance frameworks for self-learning financial agents and collaborate with policymakers to design regulatory guidelines for the responsible integration of these agents. The goal should be to strike a balance between innovation and accountability, ensuring that autonomous quantum agents support stability and fairness within the financial systems (Olubusola Odeyemi and Ekene Ezinwa Nwankwo, 2024). From a TOE perspective, technological progress such as explainable AI tools, organisational practices such as ethical review and monitoring and governance norms that promote fairness and responsible automation can help this quadrant mature beyond rapid experimentation (Table 4).

**Table 4:** Summary mapping of STM topics, trends, and framework alignment.

Datatopia quadrant	STM topic	Kendall’s τ	*p*-value	Temporal trend	Dominant TOE driver	Interpretive rationale
Society, Inc. (Controlled–Conflicted)	Quantum Interest Rates and Derivatives	−0.602	0.000	Significant decline	Environment	A mature and application-oriented research stream operating under strict regulatory and institutional constraints. The significant negative trend suggests theoretical saturation and practical limitations under existing market standards.
Aspiring Creativity (Controlled–Connected)	Quantum Market Modeling	−0.216	0.208	No significant trend	Technology	A foundational, theory-driven domain with stable attention over time. The non-significant trend indicates conceptual maturity rather than decline, consistent with controlled exploratory research.
Digital Wild West (Amok–Conflicted)	Quantum Transactions and Optimization	0.415	0.014	Significant growth	Technology	An experimental and fragmented research space characterized by rapid algorithmic development and limited standardization, reflected in a positive and significant growth trend.
Digital Wild West (Amok–Conflicted)	Quantum Risk and Management Models	0.696	0.000	Strong growth	Technology	A rapidly expanding area driven by quantum-enabled risk analytics and optimization under low institutional control, indicating strong innovation momentum.
Sorcerer’s Apprentice (Amok–Connected)	Quantum ML and Prediction	0.462	0.006	Significant growth	Technology	An emerging, highly interconnected research stream where quantum computing converges with AI, showing strong upward momentum and increasing autonomy.
Sorcerer’s Apprentice (Amok–Connected)	Quantum Trading and Agent Systems	0.462	0.006	Significant growth	Technology	A fast-growing application-oriented domain characterized by autonomous, agent-based quantum trading systems operating in highly connected digital environments.

### 5.5 Research gaps

Despite the progress in quantum finance research, several gaps remain when assessed through the TOE and Datatopia frameworks. First, organizational readiness is one such gap, as most studies focus on technological feasibility rather than institutional capacity, skills, and governance requirements for adoption. Second, the Datatopia framework has revealed a clear gap in governance and ethical considerations, with limited attention given to transparency, accountability, explainability, and regulatory oversight as quantum financial systems became more autonomous. Third, the shift of quantum financial systems toward autonomy has revealed a conceptual gap related to market stability, systemic risk, and the limits of human control. Together, these gaps have pointed to the need for a more comprehensive future research.

## 6 Implications

### 6.1 Theoretical implications

This study offers several theoretical implications for researchers. First, a gradual shift was identified in the field from theoretical models to a more applied form of research, while using the MK trend test with topics like “Quantum ML and Prediction,” “Quantum Risk and Management Models,” “Quantum Trading and Agent Systems” serving as clear indicators of this shift by having significant upward trends.

Second, our topic proportion analysis showed that AI-oriented themes are becoming influential, hinting at becoming the future research direction of the field. Third, dendrogram clustering patterns indicate that topics were clustered into groups, revealing that even though the field is fragmented, it is still connected through shared methodological foundations. Better risk management and wider use of advanced tools can contribute to long-term stability and resilience. Fourth, placing the topics in Datatopia quadrants has proved that conceptually oriented themes coexist with the technologically advanced ones, reflecting the field development. Fifth, the TOE perspective helps in understanding how technological advances in computing, organisational readiness, and environmental conditions that support experimentations and adoptions of new technologies indicate that field development is based on broader systemic influences.

### 6.2 Practical and policy implications

Our review revealed several gaps, such as a lack of interoperability, insufficient empirical evidence, and the lack of structured ethical frameworks. To address these gaps, future research should be directed toward hybrid quantum–classical experiments using real financial data and should support strong interdisciplinary collaboration. Organisational readiness must be carefully assessed through their infrastructure, skilled workforce, and governance structures of the institutions before quantum finance is implemented. Such evaluations may be facilitated through regulatory sandboxes and pilot studies. Third, the research highlights the importance of policy and ethical frameworks designed to be tailored to quantum–AI financial ecosystems to ensure that innovation advances within transparent and accountable boundaries by the regulators and recommends creating multidisciplinary regulatory task groups to jointly create interoperability, fairness, and transparency requirements. To avoid regulatory asymmetries and guarantee fair access to quantum computing equipment, international collaboration in quantum finance governance is also required (Atadoga et al., 2024).

### 6.3 Limitations and future research

This study has several limitations that should be acknowledged. First, it is based on a corpus of 81 peer reviewed documents collected from Scopus and WoS databases. Even though the two databases have wide coverage, the study may exclude significant works indexed in other databases or repositories, including pre-publication platforms such as *arXiv*. Accordingly, the findings should be interpreted as reflecting established and consolidating research trends in quantum finance rather than the full frontier of ongoing or unpublished research. We acknowledge that corpus size remains the primary limitation of the present study, and the findings should be interpreted as exploratory insights into the evolving structure of quantum finance rather than as an exhaustive representation of the entire literature.

Second, the STM model identifies latent thematic structures in an exploratory and probabilistic manner, and the interpretability of topics depends on the quality of the textual data and the preprocessing decisions adopted. As a result, STM findings should be interpreted as indicative thematic patterns rather than definitive classifications.

Third, the MK trend test identifies only the monotonic temporal trends of the topic prevalence and does not account for the cyclical or non-linear trends that might shape future research. Consequently, the identified trends reflect broad directional changes over time, rather than precise predictions of future research trajectories. Fourth, the study is based on purely secondary publication data, which limits the study and future research could benefit from incorporating primary data, such as institutional surveys and case-based analyses, to enhance and corroborate these findings. Finally, although the TOE-Datatopia framework provides a comprehensive overview of TOE factors, important emerging dimensions like sustainability, ethics and geopolitics are not fully considered into account and warrant deeper investigation in future studies.

Finally, the TOE–Datatopia framework is applied as an interpretive structure to contextualize the STM findings. Empirical alignment is achieved through a qualitative mapping of topics to dominant TOE drivers, rather than through a formal analytical integration, and the resulting interpretations should therefore be understood as indicative rather than definitive.

## 7 Conclusion

In this study, we analysed the evolution of the quantum finance field using the TOE -Datatopia framework, Mann-Kendall trend analysis and Structural Topic modelling (STM) frameworks. The MK trend test has revealed a clear change in focus from the theoretical models to more practical, applied forms of research consistent with the increasing adoption of AI in finance. A progression in the field from isolated innovation to interdisciplinary collaboration is identified through the clustering of the topics. When analysed through the TOE–Datatopia lens, the shift is shaped collectively by institutional readiness, technological development, and environmental conditions. In addition to a theoretical contribution, our study provides a structured method for examining the interdisciplinary field development by combining trend analysis with conceptual mapping. These findings can guide future work on institutional implementation, ethical design, and governance of quantum finance. Overall, our results can help policymakers and financial institutions to establish governance and ethical frameworks that align with technological progress and support the responsible deployment of AI-driven financial technologies.

## Data Availability

The data analyzed in this study is subject to the following licenses/restrictions: the data for this study were retrieved from Scopus and Web of Science, and the search terms used for data retrieval are provided within the article text for replication. Requests to access these datasets should be directed to vivekraj.s@vit.ac.in.
